# Endovascular Rescue Treatment for Delayed Cerebral Ischemia After Subarachnoid Hemorrhage Is Safe and Effective

**DOI:** 10.3389/fneur.2019.00136

**Published:** 2019-02-21

**Authors:** Miriam Weiss, Catharina Conzen, Marguerite Mueller, Martin Wiesmann, Hans Clusmann, Walid Albanna, Gerrit Alexander Schubert

**Affiliations:** ^1^Department of Neurosurgery, RWTH Aachen University, Aachen, Germany; ^2^Department of Diagnostic and Interventional Neuroradiology, RWTH Aachen University, Aachen, Germany

**Keywords:** angioplasty, delayed cerebral ischemia, endovascular rescue treatment, neuromonitoring, nimodipine, spasmolysis, subarachnoid hemorrhage, vasospasm

## Abstract

**Background:** The implementation of rescue efforts for delayed cerebral ischemia after aneurysmal subarachnoid hemorrhage remains largely empirical for a lack of supporting evidence, while the associated risk profile is also unclear.

**Objective:** The present study evaluates the safety and efficacy of endovascular rescue treatment (ERT, continuous intraarterial nimodipine; IAN, transcutaneous balloon angioplasty, TBA).

**Methods:** In this prospective observational study, we assessed periprocedural complications and side effects in context of ERT. We evaluated neurological status, multimodal neuromonitoring (p_ti_O_2_, lactate/pyruvate ratio, transcranial doppler), and cranial imaging (CTP, DSA). All parameters were included into multivariate analysis to determine predictors for the need of retreatment.

**Results:** We included 33 consecutive patients with 54 ERT (IAN *n* = 35; TBA *n* = 13; TBA + IAN *n* = 6). We recorded no serious complications and initial improvement in all parameters (neurostatus 72.3% of patients; p_ti_O_2_ 15.0 ± 11.7 to 25.8 ± 15.5 mmHg, *p* < 0.0001; lactate/pyruvate ratio 46.3 ± 27.5 to 31.0 ± 9.7, *p* <0.05; transcranial doppler 139.0 ± 46.3 to 98.9 ± 29.6 cm/s, *p* < 0.05; CTP 81.6% of patients; DSA 93.1% of patients). Retreatment (*n* = 16, 48.5%) was independently associated with preinterventional p_ti_O_2_ < 5 mmHg (*p* <0.01) and early (<72 h) discontinuation of IAN treatment (*p* = 0.08). DCI related cerebral infarction was noted in *n* = 8 patients (24.2%). At 3 months after discharge, favorable outcome was noted for *n* = 11 (35.5%) patients.

**Conclusion:** Provided a detailed decision tree, timely ERT can provide a relatively safe and effective treatment option in those highly-selected patients undergoing multimodality monitoring where conservative treatment options are exhausted. Continuous treatment in particular may be suitable to surpass sustained DCI and was associated with a low rate of DCI related infarction and comparably high percentage of good outcome.

## Introduction

Devastating outcome after aneurysmal subarachnoid hemorrhage (aSAH) is closely linked to the initial extent of injury but delayed complications may aggravate this cerebral crisis (1). In light of increased posthemorrhagic energy demand, vasospasm, and other yet unclear mechanisms frequently exacerbate metabolic and oxygenation mismatch potentially leading to cerebral infarction (2). This accumulation of detrimental mechanisms, for which delayed cerebral ischemia (DCI) stands as an umbrella term, may result in permanent neurological impairment if refractory to treatment (1).

Current pharmacological treatment options include prophylactic oral application of nimodipine (3) and vasopressor-induced hypertension as a first-line, conservative measure, though this concept has recently been challenged (4). Some patients develop persistent, refractory hypoperfusion despite conservative treatment efforts. In view of potentially catastrophic consequences when severe hypoperfusion is not addressed in a timely fashion, ERT is considered on an individual basis as a measure of last resort, even in the absence of high-level evidence.

Two principal techniques have been established in the context of endovascular rescue treatment (ERT). Proximal vessel narrowing detected on digital subtraction angiography (DSA) can be addressed via transcutaneous balloon angioplasty (TBA) but poses the risk of dissection or rupture and can result in permanent vessel wall alteration with dysfunction of autoregulation of unclear consequences (5, 6). Distal vessel constriction – inaccessible to TBA – is frequently treated with intraarterial application of vasodilating substances, supposedly reaching higher local concentrations (7). However, in addition to the risk of vessel injury, thrombosis or hemorrhage from prolonged catheterization, continuation of treatment is often limited due to its systemic hypotensive effect.

Given the considerable risk potential of ERT, efforts to identify those patients most likely to benefit from aggressive treatment and those at highest risk for treatment failure are essential. At the same time, few centers follow preset treatment algorithms to offer ERT on a regular basis. As a consequence, pivotal points to direct ERT for this particular subset of patients could not be identified so far. It is the aim of this study to provide a comprehensive assessment of the actual risk profile and present efficacy data of ERT in a center where rescue treatment is offered based on a standard operating procedure.

## Materials and Methods

### Study Design

We prospectively recruited all patients older than 18 years treated for aSAH at our institution between 01/2014 and 08/2018. Diagnosis was verified using CT and CT angiography or, if inconclusive, via lumbar puncture. For ERT analysis, we included all patients requiring at least one ERT (continuous intraarterial nimodipine (IAN), TBA) according to our standard operating procedure (see below). Written, informed consent was acquired from all patients or their respective legal representatives. This study was authorized by the local ethics committee.

### Treatment Protocol

**Figure 1:**
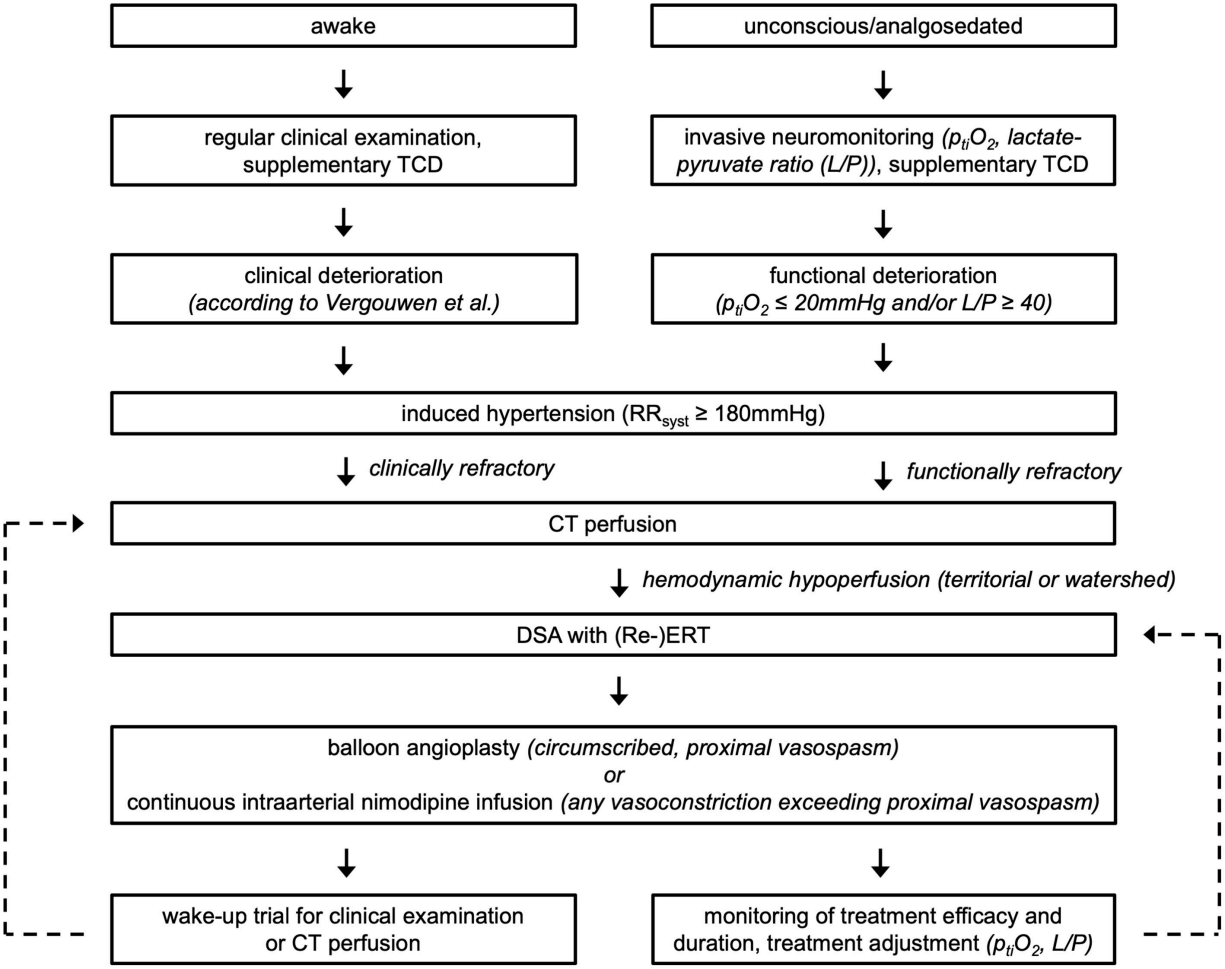
Summary of the applied diagnosis and treatment algorithm for aSAH patients at our institution.

Following aneurysm occlusion, all patients were closely monitored on a designated neurointensive care unit with clinical examinations and daily transcranial doppler sonography (TCD) (Figure 1). In patients where clinical examination was precluded (i.e., comatose and/or analgosedated), brain tissue oxygen (p_ti_O_2_) (Neurovent PTO, Raumedic, Helmbrechts, Germany), and cerebral microdialysis catheters (71 High Cut-Off Brain Microdialysis Catheter, μdialysis, Stockholm, Sweden) were inserted unilaterally into the territory carrying the ruptured aneurysm. Microdialysis catheters were perfused at 0.3 μl/min with standardized electrolyte perfusion fluid; dialysates were analyzed in 3-h intervals with shorter sampling intervals available on an as needed basis.

DCI was diagnosed according to the following criteria set by Vergouwen et al. (8): new focal neurological deficit or decrease in Glasgow Coma Scale ≥2 for a duration ≥1 h or reversible after treatment and not ascribable to other reasons. The aforementioned definition was expanded to include unconscious patients: events with functional deterioration, namely oxygenation crisis (p_ti_O_2_ < 20 mmHg), metabolic derangement as determined by cerebral microdialysis (lactate/pyruvate ratio ≥ 40) or characteristic hypoperfusion on CT perfusion (CTP), precluding any competing causes (e.g., infection, hydrocephalus) were also counted as DCI.

If DCI was suspected, systolic arterial blood pressure was raised to ≥180 mmHg using noradrenaline infusion. If clinical and functional improvement was not observed, relevant hypoperfusion was verified via CTP, followed by DSA to confirm flow limiting vasoconstriction and to direct ERT. For TBA of proximal intracranial vessel segments, Gateway or Maverick balloons (Stryker Neurovascular, Fremont, USA) were used. A bolus of nimodipine (1–2 mg/h) was applied as needed to optimize endovascular accessibility. For continuous vasodilator administration, microcatheters (Echelon^TM^10/14, Covidien/ev3/Medtronic, Irvine, USA; Rebar^TM^18, Covidien/ev3, Irvine, USA; Excelsior® SL-10®, Stryker Neurovascular, Fremont, USA) were positioned in up to three locations depending on the angiographic findings (internal carotid arteries, one vertebral artery) and separately perfused with heparinized saline solution (target partial thromboplastin time 50–60 s) and nimodipine (Carinopharm, Elze, Germany; initial dose 1–2 mg/h) at a total rate of 20 ml/h over opaque infusion lines. Analgosedation, induced hypertension, minimal handling protocol, and weight-adapted intravenous application of tirofiban (Aggrastat®, Correvio, Heathrow, United Kingdom) were maintained until termination of treatment. Stepwise de-escalation of ERT was initiated as soon as possible, guided by a combination of neurological examination, multimodal neuromonitoring and perfusion imaging. Routine clinical follow-up included assessment according to the Glasgow Outcome Scale at 3 months after discharge.

### Treatment Groups

Events of ERT were stratified into groups according to treatment modalities: TBA only, IAN only (≥12 h continuous IAN), TBA + IAN; analysis of multiple ERT per patient was permitted. Safety and efficacy parameters were documented for the total cohort and with respect to these subgroups.

### Safety Assessment

Primary safety criteria were assessed as complications related to the endovascular procedure itself and the microcatheter remaining *in situ* (dissection, perforation or thrombus formation during angiography, thromboembolic infarction during microcatheter treatment and up to 48 h after catheter removal, microcatheter occlusion with or without renewal).

Secondary safety criteria included all complications related to antiaggregation and anticoagulation (any intracranial hemorrhage) or noradrenaline demand (excessive demand necessitating a reduction of target pressure or nimodipine dose, cardio-pulmonary instability, renal or gastrointestinal impairment, peripheral microcirculatory dysfunction, i.e. cyanosis or necrosis). Comparable complications of other etiology (e.g. infection) were disregarded for this analysis.

### Efficacy Assessment

Efficacy of ERT was ascertained within 24 h after initiation of treatment: improvement of neurological examination, TCD, oxygenation (p_ti_O_2_) and metabolism (lactate/pyruvate ratio), improvement of hypoperfusion on CTP, and reversal of vasoconstriction on DSA. Cerebral infarction secondary to DCI was determined by a blinded neuroradiologist on CT imaging within 6 weeks after SAH (8). Clinical outcome was deemed favorable for Glasgow Outcome Scales 4–5 at 3 months after discharge.

### Retreatment

Retreatment was defined as repeated or additional ERT due to refractory hypoperfusion on CTP. The following potential predictors for the need of retreatment were analyzed: demographic variables (age, sex, body mass index, arterial hypertension, smoking, Hunt and Hess grade, modified Fisher grade, intracerebral, or intraventricular hemorrhage), preinterventional 24-h values of monitoring parameters (p_ti_O_2_, lactate/pyruvate ratio, TCD: means, established clinical cut-offs), vasospasm configuration (localized = proximal or distal, generalized = both), and IAN dose, coerced dose reductions in response to high noradrenaline demand, and treatment duration.

### Statistical Analysis

Statistical analysis was performed with IBM SPSS Statistics 24 (SPSS Inc., Chicago, IL, USA). Statistical significance was defined as two-sided *p* < 0.05. Parameters were dichotomized according to mean or median values for parametric or non-parametric data, respectively. Pre-post comparisons were made using paired *t*-test or Wilcoxon rank-sum test. Chi^2^ test and unpaired student's *t*-test were applied as appropriate.

To assess predictive properties for retreatment, the abovementioned variables were included into univariate analysis and, after accounting for collinearity and exclusion of all variables with *p* > 0.05, into multivariate binary logistic regression analysis.

## Results

In 33 consecutive patients (20.1% of all aSAH patients during the recruitment period), a total of 54 ERT was performed, appertaining to the modalities as follows: TBA: *n* = 13 (24.1%), IAN: *n* = 35 (64.8%), TBA + IAN: *n* = 6 (11.1%). Baseline characteristics of the study population are summarized in Table 1. ERT was started at 8.7 ± 3.5 days after hemorrhage and 3.4 ± 3.3 days after initiation of induced hypertension. IAN was started at a mean dose of 23.9 ± 8.0 μg/kg/h and maintained at a mean dose of 18.5 ± 7.5 μg/kg/h for 5.0 ± 3.4 days. Ten DCI related cerebral infarctions were noted in eight patients (24.2%). Out of 31 (93.9%) patients with available follow-up, clinical outcome at 3 months was favorable for eleven (35.5%) patients. Two patients with GOS 3 at discharge were lost to follow up. With inclusion of these two cases as unfavorable outcomes, favorable outcome amounted to 33.3%.

**Table 1:** Frequency distribution of demographic, multimodal neuromonitoring, and treatment variables with univariate and multivariate analysis results of predictors for the need of retreatment.

**Parameter**	**Frequency, *n* (%)**	**Univariate**		**Multivariate**	
		**OR (95% CI)**	***p*-value**	**OR (95% CI)**	***p*-value**
Age > 53 (mean)	16 (48.5%)	1.688 (0.414–6.878)	0.47	–	–
Female sex	22 (66.7%)	1.600 (0.369–6.946)	0.53	–	–
Body mass index > 25 (mean)	17 (51.5%)	1.004 (0.983–1.025)	0.72	–	–
Arterial hypertension	12 (36.4%)	1.750 (0.376–8.140)	0.48	–	–
Smoking	11 (33.3%)	0.530 (0.116–2.422)	0.41	–	–
Hunt and Hess grade 4–5	6 (18.2%)	0.857 (0.145–5.064)	0.87	–	–
Modified Fisher grade 3–4	22 (66.7%)	1.600 (0.369–6.946)	0.53	–	–
Intracerebral hemorrhage	10 (30.3%)	0.556 (0.122–2.536)	0.45	–	–
Intraventricular hemorrhage	18 (54.6%)	3.667 (0.849–15.844)	0.08	–	–
Anterior aneurysm location	28 (84.9%)	1.875 (0.268–13.094)	0.53	–	–
Aneurysm occlusion with clipping	16 (48.5%)	0.889 (0.216–3.662)	0.87	–	–
**LACTATE/PYRUVATE RATIO**					
>30	16 (69.6%)	1.333 (0.176–10.120)	0.78	–	–
>40	10 (43.8%)	3.000 (0.525–17.159)	0.22	–	–
>41 (mean)	7 (30.4%)	7.500 (1.039–54.116)	0.05	–	–
**p_ti_O_2_**					
< 5 mmHg	10 (25.6%)	31.500 (3.299–300.790)	0.003^*^	32.664 (2.563–416.327)	0.007^*^
< 10 mmHg	17 (43.6%)	2.625 (0.681–10.123)	0.16	–	–
< 15 mmHg (mean)	21 (53.9%)	1.250 (0.330–4.731)	0.74	–	–
**TCD**					
>120 cm/s	16 (59.3%)	0.500 (0.102–2.460)	0.39	–	–
>139 cm/s (mean)	16 (59.3%)	0.500 (0.102–2.460)	0.39	–	–
>150 cm/s	13 (48.2%)	0.750 (0.159–3.532)	0.72	–	–
>180 cm/s	7 (25.9%)	0.545 (0.095–3.146)	0.50	–	–
Generalized CVS	25 (46.3%)	4.922 (1.508–16.065)	0.008^**^	–	–
**I.A. NIMODIPINE**					
Dose >19 μg/kg/h (mean)	19 (46.3%)	2.500 (0.682–9.164)	0.17	–	–
Coerced dose reduction	17 (42.5%)	1.969 (0.542–7.145)	0.30	–	–
Discontinuation < 72 h	13 (31.7%)	5.630 (1.200–26.414)	0.03^*^	7.973 (0.766–83.046)	0.08

* *Statistically significant result*.

** *Omitted from multivariate analysis due to association with p_ti_O_2_ < 5 mmHg*.

*OR, odds ratio; CI, confidence interval; TCD, transcranial doppler; CVS, cerebral vasospasm; i.a., intraarterial*.

### Primary Safety Criteria

We recorded no vessel dissection or perforation during any intervention. In two cases (4.2%, TBA, TBA + IAN), intrainterventional thrombi were dissolved with short-term tirofiban infusion. Small, postinterventional (< 48 h) infarction was noted in three cases (6.3%, thromboembolic: *n* = 2 (TBA + IAN), periinterventional temporary occlusion due to aggravated vasospasm: *n* = 1 (TBA)). After IAN or TBA + IAN, a total of 13 microcatheters (31.7%) occluded before anticipated removal after a mean time of 3.6 ± 2.5 days. There was no case of thromboembolic infarction with an indwelling microcatheter.

### Secondary Safety Criteria

Intracranial hemorrhages during application of heparin and tirofiban were observed after four procedures (7.4%) but were limited to small hemorrhages around the trajectory of an external ventricular drain in three cases, and a small subdural hematoma treated conservatively.

Initiation of IAN resulted in a significant increase in noradrenaline requirements to maintain induced hypertension (0.4 ± 0.5 to 0.7 ± 0.6 μg/kg/h, *p* < 0.001); the increase in noradrenaline attributable to TBA did not reach statistical significance (0.3 ± 0.2 to 0.6 ± 0.4, *p* = 0.08). After IAN or TBA + IAN, excessive noradrenaline demand frequently required reduction of intraarterial nimodipine dose (*n* = 17, 42.5%), while target blood pressure could not be maintained in four cases (10.0%). Peripheral microcirculatory disturbance was observed in twelve (30.0%) patients from this subcohort which recovered following dose adjustment, while persistent decreased bowel function or paralytic ileus (*n* = 4, 10.0%), sinustachycardia or episodes of self-limiting ventricular tachycardia (*n* = 4, 10.0%), pleural effusion (*n* = 1, 2.5%), or acute kidney failure (*n* = 1, 2.5%) were less frequent. The occurrence of systemic side effects was not associated with prolonged hospitalization (42.0 ± 16.9 days with side effects vs. 38.6 ± 16.2 days without side effects, *p* = 0.41) or worsening of clinical outcome (40.0% favorable with side effects vs. 28.6% favorable without side effects, *p* = 0.52).

### Treatment Efficacy

Detailed results are summarized in Table 2. Out of eleven patients with neurological deterioration (100% of clinically evaluable patients), symptom reversal was recorded in eight patients (72.3%) after termination of analgosedation, while two patients (18.2%) did not improve and one patient (9.5%) died before regaining consciousness. Improvement rates of neurostatus, CTP and DSA were comparable for all treatment modalities. Significant improvement was seen in oxygen saturation (p_ti_O_2_), most pronounced for IAN and IAN + TBA. Overall, lactate/pyruvate ratio and TCD decreased significantly but the significance level was not reached when stratifying according to treatment modalities.

**Table 2:** Parameters of treatment efficacy for the total cohort and stratified by treatment modality.

	**Total interventions (*n = 54*)**			**TBA only (*n = 13*)**			**IAN only (*n = 35*)**			**TBA + IAN (*n = 6*)**		
**Parameter**	**pre**	**post**	***p***	**pre**	**post**	***p***	**pre**	**post**	***p***	**pre**	**post**	***p***
p_ti_O_2_, mmHg	15.0 ± 11.7	25.8 ± 15.5	< 0.0001^*^	21.5 ± 14.1	25.9 ± 17.8	0.48	13.3 ± 10.6	24.4 ± 14.5	< 0.0001^*^	11.1 ± 10.0	35.8 ± 16.8	0.07
Lactate/Pyruvate ratio	46.3 ± 27.5	31.0 ± 9.7	0.03^*^	37.7 ± 14.1	28.2 ± 4.2	0.11	41.9 ± 23.1	31.1 ± 10.5	0.30	34.7 ± 10.8	29.2 ± 10.1	0.66
TCD, cm/s	139.0 ± 46.3	98.9 ± 29.6	0.02^*^	–			128.9 ± 43.8	102.8 ± 31.7	0.16	152.0 ± 35.6	100.0 ± 20.0	0.10
Neurostatus improved, *n* (%)	8 (72.3%)			4 (80.0%)			4 (66.7%)			–		
CTP improved, *n* (%)	40 (81.6%)			10 (76.9%)			26 (83.9%)			4 (80.0%)		
DSA improved, *n* (%)	27 (93.1%)			11 (84.6%)			10 (100%)			6 (100%)		

* *Statistically significant result*.

*TBA, transcutaneous balloon angioplasty; IAN, continuous intraarterial nimodipine; TCD, transcranial doppler; CTP, CT perfusion; DSA, digital subtraction angiography*.

### Retreatment

A total of 1.6 ± 0.8 interventions were required in patients refractory to induced hypertension. Single ERT was sufficient to treat DCI refractory to induced hypertension in 17 patients (51.5%). Twelve patients (36.4%) required ERT twice and four patients (12.1%) with severe, refractory course underwent three or four ERT. According to treatment modality, retreatment was necessary as follows: IAN: *n* = 9 (25.7%), TBA: *n* = 6 (46.2%), IAN + TBA: *n* = 5 (83.3%).

**Figure 2:**
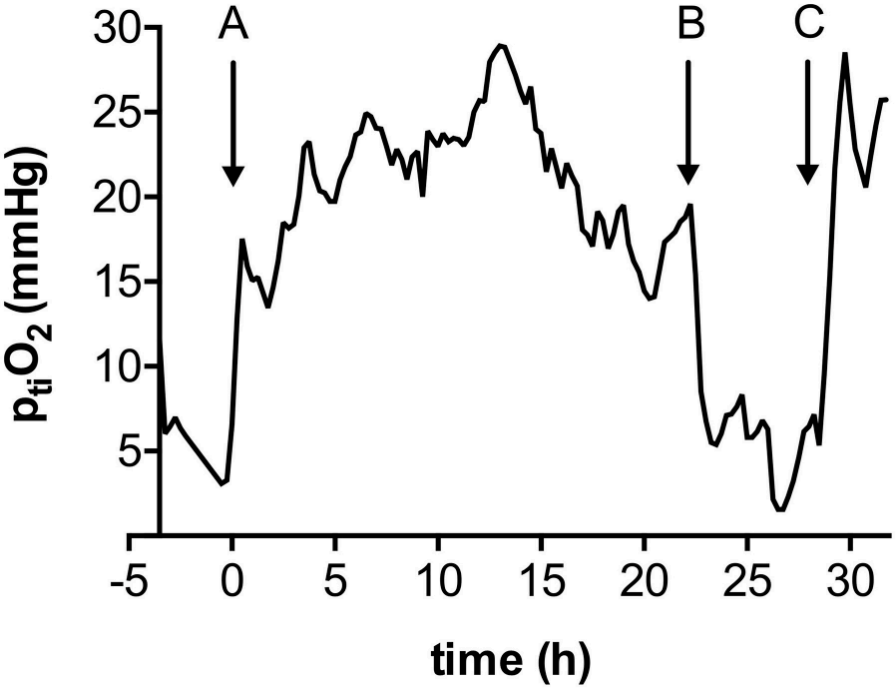
Local p_ti_O_2_ in a case of early (< 72 h) intraarterial nimodipine discontinuation. A: Initiation of IAN through a microcatheter in the right internal carotid artery; B: Occlusion of the microcatheter; C: Re-initiation of IAN through re-inserted microcatheter in the same location.

Reduction of IAN dose (1 mg/h) to maintain induced hypertension was not associated with a higher rate of retreatment compared to full dose administration (2 mg/h) (29.4 vs. 39.1%, *p* = 0.52) (Table 1). Out of 14 cases where retreatment was required after IAN or TBA + IAN, additional microcatheter placement under ongoing treatment was required only once (7.1%). In the remaining 13 cases (92.9%), IAN had been previously discontinued, resulting in secondary re-deterioration (Figure 2). The predominant reason was premature discontinuation due to microcatheter occlusion (*n* = 7, 50.0%), intentional treatment termination in three cases (21.4%), excessive noradrenaline demand (>2 μg/kg/h) in two cases (14.4%), and refractory intracranial pressure elevation in one case (7.2%).

**Figure 3:**
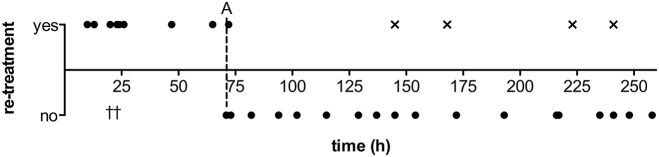
Duration of intraarterial nimodipine treatment with respect to retreatment necessity. A: Illustrative cut-off at 3 days treatment duration, indicating a frequent necessity for retreatment when discontinued prior to completion of this time window; †: Two patients in whom life-supporting measures were suspended due to poor overall condition did not receive retreatment after early (< 72 h) discontinuation; × : Out of four patients requiring retreatment after late discontinuation, three patients represent cases with severe, refractory course with 3–4 treatments or, in one case, an elongated hypoperfusion phase of 18 days, and one patient's reperfusion of the anterior territories was complicated by unilateral hypoplasia of an anterior cerebral artery.

Duration of ERT was associated with treatment efficacy: in the majority of cases (75.0%) with IAN infusion >72 h, single treatment was sufficient (Figure 3). In contrast, retreatment was required significantly more often in cases where IAN was discontinued early (72.7%, < 72 h) (*p* < 0.05). Out of ten DCI related infarctions, two (20.0%) developed following early discontinuation, while the remaining eight (80.0%) had occurred already prior to treatment with ERT.

In addition to early treatment discontinuation, intraventricular hemorrhage, preinterventional p_ti_O_2_ < 5 mmHg and generalized vasospasm were associated with a higher rate of retreatment in univariate analysis (Table 1). After multivariate analysis, only p_ti_O_2_ < 5 mmHg remained statistically significant.

## Discussion

### Safety

We experienced no procedure-related complications and only few cases of small, asymptomatic thromboembolic infarction in our cohort. Although dramatic complications have been described with ERT (5), more recent studies report a significantly lower rate of complications, with most patients recovering without clinically relevant sequelae (6, 9–12). Likewise, the rate of infarction or hemorrhage with an indwelling microcatheter has been reported as minimal (9–11), and consequently the risk of serious complications with ERT in the hands of experienced neurointerventionalists may be considerably lower than previously anticipated.

Our most common observation with induction of IAN was an increase in vasopressor demand. Although generally well-tolerated, minor adverse events were condoned in favor of brain tissue salvation. Systemic adverse events in context of excessive demand, although rare, are serious complications and must be monitored closely when implementing ERT (13), sometimes necessitating rapid reduction of intraarterial nimodipine dose to curb noradrenaline demand. In a previous study, we found nimodipine serum concentrations during intraarterial application to be dose-independent (14), but the minimum effective dose is still unclear. In the present cohort, a dose reduction was not associated with an increased rate of retreatment. In conjunction, these findings endorse the notion that immediate treatment efficacy may not correlate directly with the given dose, and that a lower dose of continuous nimodipine may be equally effective with fewer systemic impairment, provided a quantitative functional monitoring during dose titration.

### Monitoring of Treatment Efficacy

Usually, a combination of diagnostic measures (clinical examination, TCD, CTP, DSA) is employed to detect DCI with a historical focus on detecting vasospasm. However, neither diagnostic allows for the continuous assessment necessary to detect compromise in a timely manner. Intraparenchymal measurements of p_ti_O_2_ and metabolism can quantify functional impairment directly, continuously and irrespective of the underlying mechanism. Invasiveness and limited spatial coverage are well-known limitations. However, we observed functional improvement after ERT despite spatial incongruency (15), and strategic probe placement between territories may further increase coverage. In this respect, multimodal neuromonitoring offers the potential for functional quantification both before and after initiation of ERT while, at the same time, supplementing TCD and DSA by identification of non-compromising vasoconstriction that may not warrant the expenditure of ERT.

The effective clinical and functional improvement is in line with multimodal neuromonitoring results from our smaller precursor cohort (15) and from external cohorts (9–11). However, essential outcome parameters are strongly influenced by poor baseline status, making an interpretation of given results a challenging endeavor. Bele et al. observed better clinical outcome with continuous IAN compared to conservative treatment alone (11). We noted cerebral infarction in 14% and good outcome in 42% of patients which is within the range of the most recent clinical studies despite our selection of severely affected patients (6, 9–11). However, with uniform treatment protocols not in place and the lack of a conservatively managed control cohort, an interpretation of outcome dissociated from patient characteristics is prevented at this point.

### Comparison of Treatment Modalities

The premier benefit of angioplasty lies in the durability of vasodilation (16). However, the technique cannot address distal vasoconstriction and the effect remains limited to treated segments. Retreatment in our cohort was required almost twice as often with TBA than with IAN. TBA may complement IAN for severe generalized vasoconstriction with a potentially additive effect on angiographic vasodilation (17). Following severe baseline hypoxia, we saw a similar supplementary effect with greater p_ti_O_2_ increase as compared to IAN or TBA alone. However, it must be acknowledged that, despite combined treatment, retreatment, and cerebral infarction were more frequent in this severely affected subgroup of patients where ERT efficacy may reach its limit.

IAN was used with any vasoconstriction exceeding isolated proximal stenosis. Nimodipine is thought to exert a direct neuroprotective effect in addition to flow restoration as was determined in experimental settings (18, 19). This effect may translate into extended vessel territories and even extraterritorially with systemic distribution. Although not yet fully understood, direct neuroprotection on a cellular level in addition to mechanical or pharmaceutical vasodilation may be of significant therapeutic value, addressing other pathological processes contributing to DCI. Disturbed cerebral autoregulation, cortical spreading depolarization, or microthrombosis are currently considered key aspects also contributing to DCI, though their proportional share in the full development of DCI is unclear at this point. While these effects are not directly targeted by the therapies investigated in this study, nimodipine, through its neuroprotective effect, may currently still represent the best available option to treat DCI, until the exact mechanism of action and alternative drugs are discovered.

In our cohort, this was reflected by more effective reversal of local hypoxia and metabolic derangement and by a lower rate of retreatment compared to TBA. While we found no known risk factors for vasospasm (20) to be associated with retreatment, it seems reasonable that low tissue oxygenation as a surrogate of misery perfusion could be able to detect those most severely affected patients whose risk for catastrophic misery perfusion, and therefore the risk for re-deterioration, is highest.

Notably, most retreatments occurred in context of previously discontinued nimodipine infusion, whereas failure to maintain adequate perfusion under ongoing treatment was rare. Microcatheter occlusion was observed to be the reason in half of all cases where retreatment was required, an event which has been reported before (9, 11). Exchange of syringes and perfusion fluids may occasionally be associated with reflux into the catheter system accounting for an unknown proportion of catheter occlusions.

In light of such events and the supposed risks, many centers are reluctant to treat patients with an indwelling catheter for more than several hours at a time (21). As it is, intraarterial bolus application of different vasodilators remains a common form of ERT (22) although positive effects are known to be temporary with frequent cerebral infarction (23, 24). To avoid such frequent sequelae, an alternative approach is to escalate the number of consecutive ERT (21). This approach, however, was associated with an increased rate of complications and did not effectively prevent cerebral infarction.

In our severely affected cohort of aSAH patients, few DCI related infarctions were observed with continuous IAN, with the exception of two cases where IAN was discontinued early. Overall, early discontinuation was associated with a decrease of oxygenation and significantly higher need of retreatment, comparable to bolus IAN which also frequently requires retreatment. As DCI commonly manifests as an evolving process spanning several days, we hypothesize that short-time continuous treatment may not last long enough to avert this prolonged compromise, whereas sustained perfusion rescue was achieved in 83.3% in our cohort with continuous IAN treatment >72 h, while at the same time reducing the rate of transportation, recatheterization, and cumulative doses of contrast agent and radiation (25). Provided local expertise and a low risk profile and supplemented by close supervision of treatment effects (i.e., with reliable quantification of p_ti_O_2_ and metabolism), continuous IAN may offer a more appropriate profile to avoid sustained impairment.

### Study Limitations

The number of prospectively included patients exceeds that of most studies published in this context but remains relatively small in terms of statistical power. Taking into consideration the large confidence intervals resulting from this, our results may indicate underlying connections, but larger sample sizes are needed to determine accurate odds. Further adverse events not directly related to brain tissue salvation such as local injury sustained by gaining endovascular access were omitted from the study. Acknowledging the diversity of vasodilators used in other centers, conclusions resulting from this analysis may also not be extended to non-nimodipine based ERT. Most importantly, this study is limited by its lack of a conservatively treated control group. Our data suggest a reasonable risk profile and efficacy, but only randomized comparison with control groups can ultimately clarify the benefits of ERT.

## Conclusion

Provided a detailed decision tree and treatment algorithm, timely ERT can provide a relatively safe and effective treatment option in those highly-selected patients where conservative treatment options are exhausted. Multimodality neuromonitoring may be an essential mean to enable a swift reaction to incipient or recurrent deterioration. Continuous treatment in particular may be suitable to surpass sustained DCI and was associated with a low rate of DCI related infarction and comparably high percentage of good outcome.

## Data Availability

The raw data supporting the conclusions of this manuscript will be made available by the authors, without undue reservation, to any qualified researcher.

## Ethics Statement

This study was carried out in accordance with the recommendations of the ethics committee of the Medical Faculty of RWTH Aachen University with written informed consent from all subjects. All subjects or their legal representatives gave written informed consent in accordance with the Declaration of Helsinki. The protocol was approved by the ethics committee of the Medical Faculty of RWTH Aachen University (EK 062/14).

## Author Contributions

GS and MiW were involved in the conception and design. MM and MaW conducted investigated treatments. MiW and MM acquired and processed the data. MiW, GS, and WA were involved in the statistical analysis. All authors were involved in the interpretation of data. MiW drafted the article and created illustrations. All authors critically revised the article and reviewed the submitted version of the manuscript. MiW approved the final version of the manuscript on behalf of all authors. GS and WA performed study supervision.

### Conflict of Interest Statement

The authors declare that the research was conducted in the absence of any commercial or financial relationships that could be construed as a potential conflict of interest.
